# Partnership to Develop an Inter-Disciplinary Schistosomiasis Research Training Program in Uganda

**DOI:** 10.4269/ajtmh.24-0645

**Published:** 2025-04-22

**Authors:** Damalie Nakanjako, Moses Egesa, Helen Byakwaga, Casim Umba Tolo, Anatol Maranda Byaruhanga, Prudence Beinamaryo, Grace Banturaki, Lydia Nakiyingi, Ponsiano Ocama, Moses R. Kamya, Alison M. Elliott

**Affiliations:** ^1^ Department of Medicine, School of Medicine, Makerere University College of Health Sciences, Kampala, Uganda;; ^2^ Department of Infection Biology, London School of Hygiene & Tropical Medicine, London, United Kingdom;; ^3^ Uganda Virus Research Institute, Entebbe, Uganda;; ^4^ Medical Research Council/Uganda Virus Research Institute and London School of Hygiene & Tropical Medicine Uganda Research Unit, Entebbe, Uganda;; ^5^ Department of Biology, Mbarara University of Science and Technology, Mbarara, Uganda;; ^6^ Infectious Diseases Institute, Makerere University College of Health Sciences, Kampala, Uganda;; ^7^ Vector Borne and Neglected Tropical Diseases, Ministry of Health, Kampala, Uganda

## Abstract

The WHO 2021–2030 roadmap for neglected tropical diseases (NTDs) and the 2022 Kigali Declaration urge academic research institutions to unite in combating NTDs, including schistosomiasis, and emphasize the importance of strategic partnerships to free more than 1 billion people who require interventions against NTDs. We conducted stakeholder meetings to understand the landscape of schistosomiasis research training in Uganda and the existing collaborations with research institutions in sub-Saharan Africa, Europe, the United Kingdom, and the United States. In focus group discussions (involving 33 individuals from four institutions), key challenges were summarized into four emerging themes: 1) limited physical infrastructure for schistosomiasis research and training, 2) a low critical mass of scientists with competencies in schistosomiasis research, 3) a limited scope of current schistosomiasis research, and 4) limited advocacy and community engagement for schistosomiasis control. National and international partnerships, as well as partnerships between academics and implementers, should be harnessed to establish a vibrant network for schistosomiasis research training in resource-limited settings where the disease remains endemic.

## INTRODUCTION

Partnerships for global health leadership training are essential for creating effective programs that will prepare future leaders to respond to global health threats.^1^ The WHO 2021–2030 roadmap for neglected tropical diseases (NTDs) emphasizes the need for partnerships and integrated approaches to free more than 1 billion people who currently require interventions against NTDs.^2^ Successful partnerships have established self-sustaining centers of excellence in Africa, such as the Noguchi Memorial Institute for Medical Research in Ghana, the Infectious Diseases Institute at Makerere University in Uganda, the Makerere University Infection and Immunity Uganda Virus Research Institute research and training program, and the Academic Model Providing Access to Healthcare in Kenya, to address global health challenges, including HIV/AIDS, malaria, and tuberculosis.^3^^,^^4^ Experiences from these case studies have built a strong foundation for an effective, mutually beneficial, and respectful partnership that would support a concerted response to NTDs.

In a bid to optimize partnerships to achieve sustainable health for all, we set out to map existing partnerships that could be harnessed to build capacity, country ownership, and leadership in schistosomiasis prevention, diagnosis, and treatment. In addition, we explored existing resources to support schistosomiasis research training to develop the next generation of scientists with the technical competencies required to lead schistosomiasis research and control programs in sub-Saharan Africa, where the disease is endemic.

## MATERIALS AND METHODS

To map existing schistosomiasis research partnerships, stakeholder meetings were held at each participating institution: Makerere University College of Health Sciences, Mbarara University of Science and Technology, Uganda Virus Research Institute, and the Vector Borne and Neglected Tropical Diseases Division of the Ministry of Health (VCD).

### Study procedures and participants.

Participants included institutional and departmental leaders, the research and ethics committee secretariat, trainers and trainees in relevant disciplines, the VCD team, and scientists at the NIH-funded Uganda Multidisciplinary Schistosomiasis Research Center (U-SMRC). Using a focus group discussion (FGD) guide, FGDs were held to determine available resources, including physical infrastructure, collaborations, research funding, as well as challenges and recommendations for improvement. Each FGD consisted of 8–10 participants. Qualitative data were analyzed manually to identify emerging themes.

## RESULTS

Overall, 33 (nine female) stakeholders attended the stakeholders’ meetings and FGDs.

### Partnerships for schistosomiasis research and control in Uganda.

Stakeholders’ meetings revealed an understanding of the landscape of schistosomiasis research and vector control programs in Uganda, as well as existing collaborations with several academic and research institutions in sub-Saharan Africa, Europe, the United Kingdom, and the United States of America (Table 1). Similarly, several partners have contributed to the understanding of parasitic genetics (University of Glasgow, within U-SMRC), epidemiology and social science (University of Oxford), malacology (Liverpool School of Tropical Medicine), immunology and clinical trials (Cambridge University, within U-SMRC and London School of Hygiene and Tropical Medicine [LSHTM]), treatment in preschool children, clinical trials, treatment dosing, and pharmacology (Brown University, USA, with LSHTM), as well as the epidemiology of schistosomiasis and cancer (Johns Hopkins University, USA).

**Table 1:** Existing collaborations and opportunities for schistosomiasis research training at academic research institutions in Uganda

Partners	Contribution to Schistosomiasis Research and Training
Vector Borne and Neglected Tropical Diseases Division of the Ministry of Health Uganda	Laboratory placement of graduate students, offering Master of Medical Laboratory Science - Parasitology and Entomology, Malacology and Vector Control Initiatives; Technical capacity in diagnostics, field studies, parasitology; Management of schistosomiasis in the country
Uganda Schistosomiasis Multidisciplinary Research Center	Malacology, epidemiology, immunology, parasite genetics, and community engagement
Uganda Virus Research Institute	Research on viruses, vectors, and vaccines
Pharm-Biotechnology & Traditional Medicine Center (PHARMBIOTRAC)	Funding research projects involving natural therapeutics
Texas Tech University, Health Sciences Center, School of Medicine	Schistosomiasis vaccine candidate (Sm-p80; SchistoShield) and collaboration to establish a controlled human infection model for *Schistosoma mansoni*
Royal Museum for Central Africa, Belgium	Graduate student training in malacology and parasitology, citizen science approach, and community engagement for advocacy and behavioral change; Action toward reducing aquatic snail-borne parasitic diseases
Justus Liebig University Giessen, Germany	Graduate student training in malacology and bioinformatics
University of York	Schistosome proteomics and Schistosome immunology
Aberystwyth University	Schistosome drug development and cell biology
Leiden University Medical Center	Schistosome diagnostics and Schistosome immunology; Establishing a single-sex controlled human *Schistosoma mansoni *infection model for Uganda: safety and dose finding; Capacity building and hands-on laboratory work
Makerere University College of Health Sciences	Master’s and doctoral training programs in medical microbiology, pathology, clinical epidemiology and biostatistics, immunology and molecular biology, bioinformatics, and public health
University of Cape Town	Bioinformatics
University of Notre Dame	Genomics
Center for Research in Infectious Diseases/London School of Tropical Medicine	Epidemiology, statistics, modeling, clinical trials, and pharmacology
Imperial College London	Attachment of students from Imperial College to VCD
University of Glasgow	Attachment of students from University of Glasgow

VCD = Vector Borne and Neglected Tropical Diseases Division of the Ministry of Health.

### Ongoing schistosomiasis research programs.

We identified ongoing schistosomiasis research programs that cover several aspects of schistosomiasis. Examples include efforts to understand differences in schistosomal morbidity in endemic areas with comparable transmission and treatment coverage under the U-SMRC, as well as a project aimed at understanding the clinical and immunological impact of *Schistosoma mansoni* (*S. mansoni*) infection and treatment on the course of hepatitis B virus infection among Ugandans.^5^ We also identified ongoing work to establish human infection studies for *S. mansoni* in Uganda to accelerate schistosomiasis vaccine development,^6^ along with another study examining the impact of increased praziquantel frequency on childhood fibrosis in persistent schistosomiasis morbidity hotspots.^7^^,^^8^ These projects, summarized in Table 2, along with similar projects, provide a platform for schistosomiasis research training through collaboration with local and international academic institutions to support both degree and non-degree awarding training.

**Table 2:** Ongoing schistosomiasis research projects that will complement a comprehensive schistosomiasis research training program

Research Program/Project	Schistosomiasis Area of Focus	Main Objective/Research Question of Project	Funding	Reference
Uganda Schistosomiasis Multidisciplinary Research Centre	Morbidity, human immunology, *Schistosoma* population genetics, malacology	Understanding differences in severe schistosomal morbidity in endemic areas of comparable transmission and treatment coverage	NIAID/NIH	https://tmrc-network.org/research-centers/uganda.
Clinical and immunological impact of *Schistosoma mansoni* infection and treatment on the course of hepatitis B virus infection among Ugandans	Human immunology, morbidity, diagnostics	Characterizing the nature and magnitude of impact from *Schistosoma mansoni* infection on the course of chronic HBV	NIAID/NIH	https://uvri.go.ug/.
Human infection studies for *Schistosoma mansoni* (CHI-S) in Uganda	Human challenge, vaccine trial, Immunology, malacology, stakeholder engagement, social science, ethics and regularatory aspects	Establishing CHI-S in Uganda to accelerate schistosomiasis vaccine development	Wellcome Trust	https://www.lshtm.ac.uk/research/centres-projects-groups/chi-s.
Innovations for vaccines against helminth infections	Vaccine discovery and production platforms, human challenge, vaccine trial, human immunology, molecular biology, animal models, vaccine testing (pre-clinical and Phase 1), data science	Establishing an effective pipeline to develop helminth vaccines for schisto and hookworm	Horizon Europe	https://www.wormvacs.org/.
Impact of increased praziquantel frequency on childhood fibrosis in persistent schistosomiasis morbidity hotspots	Morbidity, diagnostics, treatment, drug dosing, peadiatrics	Elucidating whether increased treatment frequency reduces the prevalence of childhood periportal fibrosis in hotspots of persistant schistosomiasis	EDCTP2	https://www.fibroschot.eu/.
*Schistosoma mansoni* protein and glycan epitope mapping	Immunology, functional glycomics, proteomics	Deciphering antibody responses to natural and controlled human infection in an endemic Ugandan population	EDCTP2	https://www.lshtm.ac.uk/research/centres-projects-groups/smchi-epitope#welcome.

CHI-S = controlled human infection model for schistosomiasis; EDCTP2 = Second European & Developing Countries Clinical Trials Partnership program; HBV = hepatitis B virus; ISPF = International Science Partnership Fund; NIAID/NIH = National Institute of Allergy and Infectious Diseases of the National Institutes of Health.

### Challenges to schistosomiasis control, as perceived by stakeholders in Uganda.

Each FGD included six to eight individuals. Key challenges reported in the FGD were summarized into four emerging themes: 1) limited physical infrastructure for schistosomiasis research and training, 2) low critical mass of scientists with competencies in schistosomiasis research, 3) limited scope of schistosomiasis research, and 4) limited advocacy and community engagement for schistosomiasis control (Table 3).

**Table 3:** Challenges facing schistosomiasis research and disease control in Uganda

Theme	Responses
Physical infrastructure for schistosomiasis research and training	Limited laboratory facilities and equipment for experiments in endemic regions
	Limited training datasets for the application of genomics and bioinformatics in schistosomiasis research
Low critical mass of scientists with competences in schistosomiasis research	Limited schistosomiasis experts
	Lack of mentors in the schistosomiasis field
	Limited opportunities for in-service training for health workers
	Limited number of socio-behavioral scientists with expertise in schistosomiasis-endemic communities
Scope of schistosomiasis research	Limited translational research and product discovery
	Limited application of genomics and bioinformatics in schistosomiasis research
	Limited application of artificial intelligence and big data in the prediction and prevention of schistosomiasis
Limited advocacy and community engagement for schistosomiasis control	Low community engagement and advocacy for schistosomiasis management, prevention, and control
	Schistosomiasis remains low on all priority lists; hence its status as a neglected tropical disease
	No media coverage on the impact of the disease and its prevention
	Limited engagement of national policymakers

To address these challenges and enhance schistosomiasis research capabilities, stakeholders recommended: 1) comprehensive training programs to equip scientists with the skills required to design research, use research data, and engage the community in adopting evidence-based interventions; 2) a focus on high-priority training areas such as One Health perspectives, diagnostic techniques, community involvement, and ensuring a holistic and relevant approach to schistosomiasis research; and 3) intentional engagement of stakeholders, including policymakers, funders, and mentors, to promote advocacy, the prioritization of funding, and a conducive environment for schistosomiasis research. These recommendations inform the development of effective and responsive schistosomiasis research training programs that can overcome challenges and meet the diverse needs of schistosomiasis researchers in Uganda.

## DISCUSSION

Global partnerships tackling NTDs will reduce poverty, address inequity, strengthen health systems, increase human capital, and build resilient communities, bringing us closer to achieving universal health coverage and the Sustainable Development Goals.^9^ Endemic countries are the beneficiaries of progress toward the WHO 2030 NTD targets and must also be the drivers, leading, synergizing, and optimizing national and global partnerships to achieve control, elimination, and eradication of NTDs, including schistosomiasis.^2^

Academic research institutions play a key role in the sustainability of research and, consequently, in the development of evidence-based health care tools and interventions. They contribute to the pipeline by training experts in specific and cross-cutting areas, including transmission patterns, epidemiology, modeling, surveillance, and molecular and immunological studies, as well as monitoring and evaluation research. Academic institutions need to be empowered to lead and optimize the integration of NTD research training, in line with the Kigali Declaration on uniting to combat NTDs.^9^ They must foster well-coordinated and collaborative action toward the eradication and elimination of NTDs.

Our focus is on schistosomiasis in Uganda. More than one-quarter of the population is estimated to be infected, and the disease remains severe along the Albertine Nile Region in the northwestern region.^10^^–^^14^ We identified several existing collaborative schistosomiasis research initiatives with academic and research institutions in Africa, Europe, the United Kingdom, and the United States, which provide opportunities for multi-sectoral research training in schistosomiasis and other NTDs. Lessons learned from other collaborative training programs in sub-Saharan Africa should be utilized to ensure 1) efficient administration to provide a conducive environment for high-quality research; 2) supportive policies for procurement, including provisions for the purchase of specific biological research reagents from international manufacturers; and 3) mentorship, which catalyzes young scientists to progress from graduate trainees to productive academic researchers focused on schistosomiasis and other NTDs.^4^ Although it has not been evaluated in this study, it is essential to ensure that partners in schistosomiasis research have a shared strategic vision and are prepared to address inequalities that may arise from disparities in infrastructure, managerial expertise, and administrative and leadership capacity, as well as to ensure mutual benefit and mutual respect.^1^

Stakeholders recommend that Schistosomiasis research training programs should aim to 1) equip scientists with the skills required to design research, use research data, and engage with the community to promote research uptake; 2) focus on high-priority training areas such as One Health perspectives, diagnostic techniques, and community involvement; and 3) promote advocacy with a prioritization of funding for the most-at-risk communities. These recommendations inform the development of effective and responsive schistosomiasis research training programs that can overcome challenges and meet the diverse needs of schistosomiasis researchers in Uganda.

Our findings are in line with the commitment of academic research institutions, as stated in the 2022 Kigali Declaration, to support multisectoral and multidisciplinary research (involving biomedical and social sciences) to tackle NTDs, including the One Health approach, which recognizes the interactions among human, animal, and environmental health, and mitigates the impact of climate change on NTDs.^9^ In addition, academic institutions pledged to advance comprehensive educational curricula on the prevention, treatment, and control of NTDs, with a focus on building the capacity and excellence of the health and research workforce. Through collaborative initiatives such as those presented in this paper, institutions should develop robust monitoring and evaluation mechanisms across sectors to measure the impact of actions aimed at controlling, eliminating, and eradicating NTDs. Guidelines and training on the evaluation of partnerships exist and should be used, including generic indicators of equity, mutual benefit, and the added value of collaboration.^1^

## CONCLUSION

We recommend harnessing all collaborating partners from academic research institutions in sub-Saharan Africa, Europe, the United Kingdom, and the United States to establish a vibrant network for schistosomiasis research and training, which is in line with the WHO 2021–2030 roadmap for NTDs and the Kigali Declaration of academic research institutions to unite and combat NTDs, including schistosomiasis.
